# APASL practical recommendations for the management of hepatocellular carcinoma in the era of COVID-19

**DOI:** 10.1007/s12072-020-10103-4

**Published:** 2020-11-11

**Authors:** Shuichiro Shiina, Rino A. Gani, Osamu Yokosuka, Hitoshi Maruyama, Hiroaki Nagamatsu, Diana Alcantara Payawal, A. Kadir Dokmeci, Laurentius A. Lesmana, Tawesak Tanwandee, George Lau, Shiv Kumar Sarin, Masao Omata

**Affiliations:** 1 Department of Gastroenterology, Juntendo University, 2-1-1, Hongo, Bunkyo-ku, Tokyo, 113-8421 Japan; 2 Department of Internal Medicine, Cipto Mangunkusumo Hospital, University of Indonesia, Jakarta, Indonesia; 3 Chiba University, Chiba, Japan; 4 Department of Hepatology, Cardinal Santos Medical Center, Manila, Philippines; 5 Department of Gastroenterology, Ankara University School of Medicine, Ankara, Turkey; 6 Digestive Disease and GI Oncology Center, Medistra, Hospital, University of Indonesia, Jakarta, Indonesia; 7 Department of Medicine, Faculty of Medicine, Siriraj Hospital, Mahidol University, Bangkok, Thailand; 8 Humanity and Health Clinical Trial Center, Humanity and Health Medical Group, Hong Kong SAR, China; 9 Liver Disease and Transplant Center, The Fifth Medical Center of Chinese PLA General Hospital, Beijing, China; 10 Department of Hepatology, Institute of Liver and Biliary Sciences, New Delhi, 110070 India; 11 Department of Gastroenterology, Yamanashi Prefectural Central Hospital, Kofu-city, Yamanashi Japan; 12 The University of Tokyo, Tokyo, Japan

**Keywords:** The novel coronavirus, Liver cancer, Asian-Pacific, Chronic liver disease, Asymptomatically infected, Hospital preparedness, Surveillance, Diagnosis, Facemask, CT screening for COVID-19, Treatment, Decision-making, Personal protective equipment, Aerosol generating procedure

## Abstract

**Background:**

COVID-19 has been giving the devastating impact on the current medical care system. There are quite many guidelines on COVID-19, but only a few on the management of hepatocellular carcinoma (HCC) during COVID-19 pandemic.

**Aims:**

We develop these recommendations to preserve adequate clinical practice for the management of HCC.

**Methods:**

Experts of HCC in the Asia–Pacific region exchanged opinions via webinar, and these recommendations were formed.

**Results:**

Close contact should be minimized to reduce possible exposure of both medical staff and patients to the novel coronavirus. To prevent transmission of the virus, meticulous hygiene measures are important. With the decrease in regular medical service, the medical staff may be mobilized to provide COVID-19-related patient care. However, diagnosis and treatment of HCC should not be delayed because of COVID-19 pandemic. The management of HCC should be the same as in non-pandemic circumstances. HCC is highly malignant, thus it is recommended not to delay curative treatment such as surgery and ablation. However, a kind of triage is necessary even among patients with HCC when resources are insufficient for all to be treated. Curative treatments should be periodized and cytoreductive or non-curative treatment such as vascular interventions and systemic therapy may be postponed until it can be performed safely with sufficient resources. For patients with confirmed or suspected to be infected with the novel coronavirus, diagnosis and treatment should be postponed until the virus is eliminated or they are confirmed as not being infected with it.

**Conclusions:**

These are collection of measures implemented by front-line medical professionals. We would evolve these recommendations over time as more real-world data becomes available.

## Introduction

Coronavirus disease 2019 (COVID-19), an infectious disease caused by the novel coronavirus (SARS-CoV-2) has brought upon devastating stress on the current medical care system. The World Health Organization (WHO) recognized the outbreak of COVID-19 as a pandemic on 11 March 2020. As of 19th Aug 2020, COVID-19 pandemic has resulted in over 21.9 million confirmed cases globally, and over 775 thousand deaths. Approximately 250 thousand newly-confirmed cases are being reported daily. The pandemic has not settled down yet [1]. The virus is mainly transmitted from person-to-person through respiratory droplets and contact with contaminated surfaces or objects. There are also other modes of transmission (i.e. airborne and fecal–oral). The average incubation period is 5–6 days, ranging from 0 to 14 days. The clinical spectrum of COVID-19 ranges from mild disease with non-specific signs and symptoms of acute respiratory illness to severe pneumonia with respiratory failure and septic shock. There are also reports of asymptomatic cases. Currently, there is no vaccine against the novel coronavirus. The best way to prevent COVID-19 is to avoid being exposed to the coronavirus. It is recommended that we should keep distance of at least 2 m from other people. A new modeling study shows that intermittent periods of social distancing strategies will be needed till 2022 to avoid overloading of the medical system with its limited critical care capacity [2].

Until the end of this COVID-19 pandemic, one needs to maintain high management standards of other diseases despite the shortage of medical resources, such as personnel, beds, personal protective equipment (PPE) and ventilators. There are a number of guidelines and recommendations on COVID-19 [3–5]. However, there are only few guidelines and recommendations on the management of hepatocellular carcinoma (HCC) on pandemic of COVID-19 [6, 7].

Currently, liver cancer is the fourth most common cause of cancer-related death in the world, which caused 782 thousand deaths in 2018 [8]. More than 90% of liver cancer is hepatocellular carcinoma (HCC). The majority of cases of HCC are found in the Asian-Pacific region [9]. Since Asia–pacific region has more experience both in HCC and in COVID-19 than anywhere else, experts of HCC exchanged opinions via webinar and developed these recommendations to preserve and sustain adequate clinical practice in the management of HCC in the era of COVID-19.

APASL has presented clinical practice guidance for general hepatology and liver transplant providers during the COVID-19 pandemic which also includes HCC [10]. Our article focuses specifically on HCC, presents more practical recommendations and elaborates on the different management needs. Our article does not have either grading of evidence or grading of recommendation as well as many recommendations concerning COVID-19 [3–7, 11]. Since COVID-19 is a novel disease, guidance by scientific evidence is rarely available. The recommendations are the collection of measures implemented by front-line medical professionals and are revised through intense interaction via webinar. The presenter, the moderators and co-authors exchanged opinions and discussed the contents via emails and phones. These recommendations are likely to evolve over time as further data become available.

## COVID-19 and cancer

Patients with cancer seem to be easier to be infected with the novel coronavirus than individuals without cancer. A prospective study in China revealed that 18 (1%) of 1590 COVID-19 patients had a history of cancer**,** which seems to be higher than the incidence of cancer in the overall Chinese population [12]. They are also likely to be at an increased risk of progression to severe diseases with COVID-19, such as being admitted to the intensive care unit requiring invasive ventilation, and death, compared with patients without cancer. A retrospective study in Wuhan, China reported that 12 (0.79%) of 1524 patients with COVID-19 had cancer. It revealed that patients with cancer from the epicenter of COVID-19 had a higher risk of the novel coronavirus infection compared with the general population. Seven (58.3%) of 12 patients had non-small cell lung cancer [13]. In a different retrospective study, there were 28 patients with cancer who were admitted for COVID-19 in three hospitals in Wuhan and who had received cancer therapy within 14 days. They were found to be associated with substantially higher risk of mortality [14]. Patients with cancer may be susceptible to the infection and more likely to have higher morbidity and mortality than the general population. Several factors could account for an elevated risk for the infection and consequent complications among cancer patients, including systemic immunocompromised state caused by the malignancy and anticancer treatments, frequent hospital visits, advanced age, and poor performance status.

Cancer is the second leading cause of death. An estimated 9.6 million people died of cancer globally in 2018, i.e., about 1 in 6 deaths was due to cancer. The five most common causes of cancer death were cancers of lung (1.76 million deaths), colorectal (862 thousand deaths), stomach (783 thousand deaths), liver (782 thousand deaths) and breast (627 thousand deaths) [8].

Cancer mortality can be reduced if cases are detected and treated without delay. When detected early, cancer is more likely to respond to effective treatment and can result in a greater probability of surviving, less morbidity, and less expensive treatment. Significant improvements can be made in the lives of cancer patients by detecting cancer early and avoiding delays in treatment. We should not delay the diagnosis and treatment of cancer in the era of COVID-19 pandemic. However, it would be necessary to tailor the management depending on available resources. Indication of any invasive procedure should be decided on a case-by-case basis with the consideration of the increased risk during the pandemic, the urgency of the procedure and the effect of the delayed intervention.

## COVID-19 and chronic liver disease

Most patients with HCC have underlying chronic liver disease, resulting from chronic hepatitis B virus (HBV) or chronic hepatitis C virus (HCV) infection, alcoholic liver disease, and non-alcoholic fatty liver disease. There have not been sufficient data on whether patients with chronic liver disease are at increased risk for getting COVID-19 or having severe COVID-19 [15]. CDC COVID-19 Response Team reported that 2692 (37.6%) of 7162 patients with full past history of illness had one or more underlying health condition or risk factor. They also reported that 41 (0.6%) had chronic liver disease, 17 (41%) of whom were hospitalized and 7 (17%) were treated in ICU [16]. Incidentally, the most commonly reported conditions were diabetes mellitus, chronic lung disease, and cardiovascular disease. In other series of COVID-19 reported by Chinese centers, 2–11% of patients had comorbid chronic liver disease [16–22]. There is a report that patients with non-alcoholic fatty liver disease were associated with COVID-19 progression [23].

Infection with the novel coronavirus may impact existing chronic liver disease in three ways: first, the additional hepatic injury induced by COVID-19 could lead to hepatic decompensation in patients with compromised hepatic reserves [24]. Second, the potential immunosuppressive properties induced by the novel coronavirus may lead to viral reactivation in patients with chronic viral hepatitis, although more data are required to confirm this hypothesis [24, 25]. Third, drugs used for the treatment of COVID-19 may produce hepatotoxicity, as suggested by histological features of moderate microvascular steatosis with mild hepatic inflammation [26, 27]. Many patients had antipyretic drugs for fever. Most antipyretic drugs contain acetaminophen, which has direct hepatotoxic potential. Furthermore, many patients with COVID-19 may be treated with novel, potentially hepatotoxic, antiviral drugs as well as antibiotics for bacterial superinfections.

## Hospital preparedness for COVID-19

It is important to minimize exposure of both medical staff and patients to the novel coronavirus. Face-to-face consultation should be performed through web consultation or by telephone call whenever possible. Hospitals should adapt strategies for patients to minimize hospital visits and hospitalization. Routine follow-up visit of hospitals should be postponed as long as possible. If circumstances allow, the appointment registration system should be adapted. Pre-reception inspection should be set up including body temperature check and Questionnaire (symptoms, recent history of close contact with confirmed or suspected person infected with the novel coronavirus). It is important to maintain social safety distance of 2 m between individuals. In general, hospitals should not allow patients to be accompanied with others because they can be asymptomatically infected with the virus.

There are various ways to screen individuals infected with the novel coronavirus. Reverse transcription polymerase chain reaction (RT-PCR) is the current standard test for COVID-19. In well-equipped hospitals, PCR assays have been conducted a few days prior to admission. Antigen test is another method of detecting the presence of the virus itself. Although it is less accurate, it offers results in about 15 min as opposed to hours for PCR. Both methods need a cumbersome swab to take samples from the cavity between the nose and mouth (nasopharyngeal swab). Another method is detection of antibodies (serological tests). Antibody tests show how many people have had the disease, including those whose symptoms are minor or who are asymptomatic. The serum antibody rises to certain level at an early period in patients infected with the virus.

In general, chest CT is not recommended for detecting the novel coronavirus infection. CT should only be deployed in very specific conditions. One is in patients who have high risk of the novel coronavirus infection [26], because the sensitivity of PCR is not 100%. Another condition is in patients who are scheduled to be hospitalized and have abdominal CT, because it is not troublesome to take chest scan simultaneously. Chest CT screening for COVID-19 has a low pick up rate in asymptomatic patients infected with the novel coronavirus and a 20% false negative rate in symptomatic patients [26, 27]. Radiologic findings in COVID-19 are not specific. Typical features on CT initially include bilateral multilobar ground-glass opacities with a peripheral or posterior distribution.

## COVID-19 and HCC

Generally, the diagnosis and treatment of HCC, which is a highly malignant tumor, should not be delayed because of pandemic of COVID-19. The indications and principles of management of HCC should be the same as in non-pandemic circumstances. However, a kind of triage may be necessary even among patients with HCC when resources are insufficient. With the decrease in regular medical service, staff may be mobilized to provide COVID-19-related patient care. This shift of staff should not have a negative impact on the ability to manage HCC patients.

Patients with HCC who have symptoms suggestive of COVID-19 or other conditions of high risk of the novel coronavirus infection, such as close contact with infected people or people who come from a severely affected area, and have not been tested should undergo PCR. If available, chest CT should also be performed for those who have high risk of the novel coronavirus infection [17, 20, 22]. For patients with confirmed or suspected to be infected with the novel coronavirus, diagnosis and treatment of HCC should be postponed until the virus is eliminated or they are confirmed as not being infected with it. It is supposed that the median time from onset to clinical recovery is approximately 2 weeks for mild cases and is 3–6 weeks for severe or critical disease cases.

## Surveillance for HCC

Generally, surveillance with ultrasound (US) and serum alpha-fetoprotein (AFP) measurement are undertaken in high-risk groups of patients, such as those with chronic HBV or HCV hepatitis and those with liver cirrhosis of any etiology. US is widely practiced for surveillance of HCC since US is noninvasive and its cost is reasonable. However, in the era of COVID-19, surveillance using US should be limited because of the close contact with patients and US practitioner [28, 29].

Under normal circumstances, a 6-month surveillance interval is considered as a standard for high-risk subjects [30]. Since COVID-19 decreases the regular medical service, it would be necessary to prioritize surveillance of super high-risk patients of developing HCC, such as those with HBV- or HCV-related liver cirrhosis.

## Diagnosis for HCC

### General principles in the era of COVID-19

In the era of COVID-19 pandemic, the diagnosis of cancers as well as other diseases would not work smoothly in many hospitals because of their limited capacity. However, the diagnosis of HCC should be prioritized because HCC is highly malignant. Every patient should be considered as possibly infected with the novel coronavirus. Table 1 summarizes recommendations for general principles and each examination for HCC.

**Table 1:** Recommendations for diagnosis of hepatocellular carcinoma during COVID-19 pandemic

Interventions	Recommendations
General principles	The diagnosis of HCC should be prioritized Every patient should be considered as possibly infected with the novel coronavirus For patients with confirmed or suspected to be infected with the novel coronavirus, diagnosis should be postponed until the virus is eliminated or they are confirmed as not being infected
Ultrasound (US)	Usage of US should be limited US practitioners should wear surgical facemasks Examinees are also advised to wear facemasks When examinees do not wear masks, US practitioners should also have eye guards
CT and MRI	In general, chest CT should not be deployed for screening of COVID-19 Those who have an abdominal CT in their investigation may also have chest CT scan at the same time Equipment, surfaces and contact points should be deeply cleaned after each examination

### US

It is recommended to limit the usage of US, since US practitioners are in close contact with patients [28, 29]. Contrast-enhanced US, which is very sensitive to detect hypervascularity in a nodule, should also be limited. As a protection for both US practitioners and patients, surgical facemasks are necessary for US practitioners. Examinees are also advised to wear facemasks. When examinees do not wear masks, US practitioners should have eye guards. Every patient should be considered as possibly infected with the novel coronavirus. To prevent transmission of the virus, meticulous hygiene measures are important. After each examination, thorough cleaning of a gel bottle and all touched surfaces should be performed using a disinfectant [28, 29].

### CT and MRI

In general, dynamic CT, dynamic MRI, or gadolinium ethoxybenzyl diethylenetriamine pentaacetic acid (Gd-EOBDTPA)-enhanced MRI is recommended as a first-line diagnostic tool for HCC when a screening US shows a possible HCC nodule [30]. As every patient possibly infected with the novel coronavirus, those who have an abdominal CT in their investigation may also have chest CT scan at the same time [31]. It would not be troublesome to take chest scan additionally.

However, chest CT should only be deployed in very specific circumstances, because CT screening for COVID-19 has a low pick up rate in asymptomatic patients infected with the novel coronavirus and a 20% false negative rate in symptomatic patients [26, 27].

After each examination, deep cleaning of equipment, surfaces and contact points is mandatory [32]. If possible, establishment of a COVID-19 dedicated CT and/or MRI scanner can minimize the risk of hospital-acquired infection.

## Treatment for HCC

### General principles

We need to decide whether we should perform, postpone or suspend treatment for each individual case by considering the situation in real time not only from medical but also from logistical viewpoint. Telemedicine is important in multi-disciplinary decision-making process on treatment and care plans. This technology would make it possible for HCC patients to get the right treatment at the right time without delay.

HCC as well as most other cancers are considered as progressive diseases, thus it is recommended not to delay their curative treatment such as surgery and ablation. American College of Surgeons recommends Elective Surgery Acuity Scale [33], which would be useful to assist the decision-making process not only of surgery but also of other actions to triage non-emergent interventions. HCC as well as most other cancers may be categorized as “Tier 3a (high acuity)”, thus action should not be postponed in general. However, a kind of triage is necessary even among patients with HCC when resources are insufficient for all to be treated without delay. Curative treatments should be more prioritized and cytoreductive or non-curative treatments may be postponed until they can be performed safely. The situation may be different from country to country and from institution to institution.

During the intervention, minimum number of staff should be in the room and all staff in the room should wear appropriate PPE [34] depending on their role and risk. In some interventions without aerosol generation, PPE may be necessary only for operators who have close contact with patients possibly infected with the novel coronavirus. Procedure tasks are slower and more difficult when wearing full PPE [35]. Table 2 summarizes recommendations on general principles and each treatment for HCC.

**Table 2:** Recommendations for treatment of hepatocellular carcinoma during COVID-19 pandemic

Interventions	Recommendations
General principles	Whether to perform, postpone or suspend treatment should be decided for each individual case not only from medical but also from logistical viewpoint It is recommended not to delay a curative treatment such as surgery and ablation Cytoreductive or non-curative treatment such as vascular interventions and systemic therapy may be postponed For patients with confirmed or suspected to be infected with the novel coronavirus, treatments should be postponed until the virus is eliminated or they are confirmed as not being infected with it
Liver resection	Generally, liver resection with curative intent should not be delayed However, in cases of high risk of decompensation or comorbidities, surgical intervention should be postponed or alternative therapy such as ablation should be adapted There has been some concern on the safety of surgery as surgery cannot be performed without aerosol generating procedures
Liver transplantation	Liver transplantation for patients with poor short-term prognosis should not be delayed Elective living donor transplantation may be suspended In patients with complete response to bridging therapy on transplant list, transplantation may be suspended
Ablation	Ablation with curative intent should not be delayed Ablation is an acceptable alternative to resection for cases of three or fewer tumors, each 3 cm or smaller, and of Child–Pugh class A or B liver dysfunction
Vascular interventions	Vascular interventions may be postponed because they are used as cytoreductive treatments in most cases Vascular interventions should be suspended in cases of risk of decompensation or comorbidities that increase the risk of severe COVID-19
Radiation therapy	Radiation therapy for cases of symptoms control or at low risk of progression may be postponed However, radiation therapy for function- or life-threatening situation have to be treated without delay The course of radiation should be shortened when appropriate
Systemic therapy	Oral tyrosine kinase inhibitors would be better than infusional regimens during the pandemic The impact of immunotherapy on the course of COVID-19 is not known

### Liver resection

Liver resection (LR) is a curative treatment for HCC among Child–Pugh class A patients, although recurrence develops in most cases even after radical resection. Its indication should be decided based on not only tumor condition but also liver function. Generally, liver resection with curative intent should not be delayed. However, in cases of high risk of decompensation or comorbidities that increase risk of severe COVID-19, surgical intervention should be postponed or alternative therapy such as ablation should be adapted.

There has been some concern on the safety of surgery during COVID-19 pandemic as surgery cannot be performed without aerosol generating procedures, such as endotracheal incubation and energy device usage which produces surgical smoke. Laparoscopic or robotic surgery during the pandemic may contribute to decreased length of stay as compared with open surgery as well as minimizing the need for medical treatments. On the other hand, pneumoperitoneum, which is inevitable in laparoscopic or robotic surgery may bring higher risk of aerosol exposure to the surgeons and staff.

### Liver transplantation

Liver transplantation, which is the best therapeutic option in some patients because it can be a treatment not only for HCC but also for cirrhosis. However, organ donor shortage restricts its indication.

During COVID-19 pandemic, transplantation should be decided on case-by-case basis. Liver transplantation for patients with poor short-term prognosis, such as with high MELD score and HCC at the upper limits of the Milan criteria are in high priority and should not be delayed [36, 37] and for those with compensated liver disease and within the lower limits of Milan criteria have medium priority may be suspended to minimize the risk of the donor and the recipient. In patients with complete response to bridging therapy on transplant list, transplantation may also be suspended until it can be performed safely with sufficient resources.

### Ablation

Image-guided percutaneous ablation, such as radiofrequency ablation, microwave ablation and others, are minimally invasive therapies for HCC. Ablation is a potentially curative treatment and easily repeatable for recurrence. Ablation with curative intent should not be delayed. In cases of three or fewer tumors, each 3 cm or smaller, and of Child–Pugh class A or B liver dysfunction, ablation is an acceptable alternative to resection [38].

**Fig. 1:**
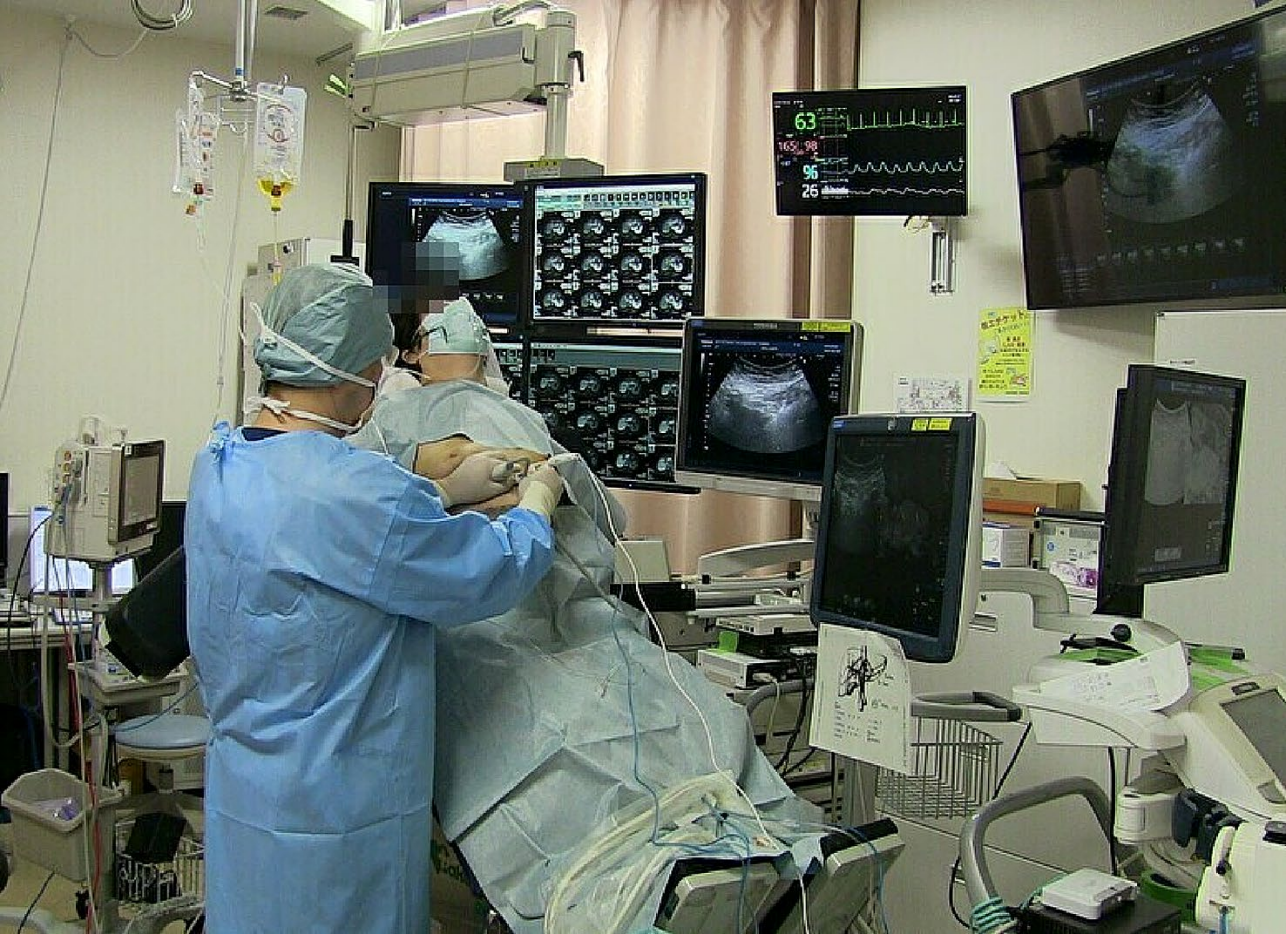
Image-guided percutaneous ablations, such as radiofrequency ablation, microwave ablation and others, are minimally invasive therapies for HCC. Ablation with curative intent should not be delayed even during the COVID-19 pandemic. The patient is in an upright position. An RFA electrode is inserted from the epigastrium into a tumor in the left lateral segment

**Fig. 2:**
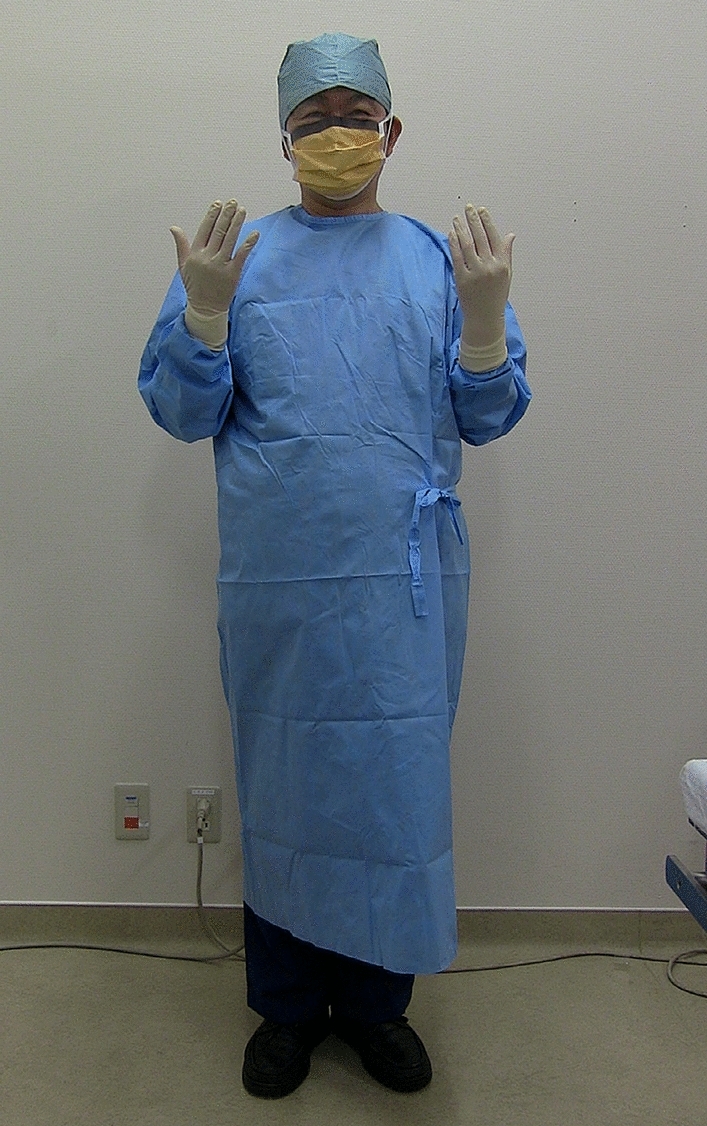
Personal Protective Equipment (PPE) consists head cover, eye guard, surgical mask, isolation gown, gloves

**Fig. 3:**
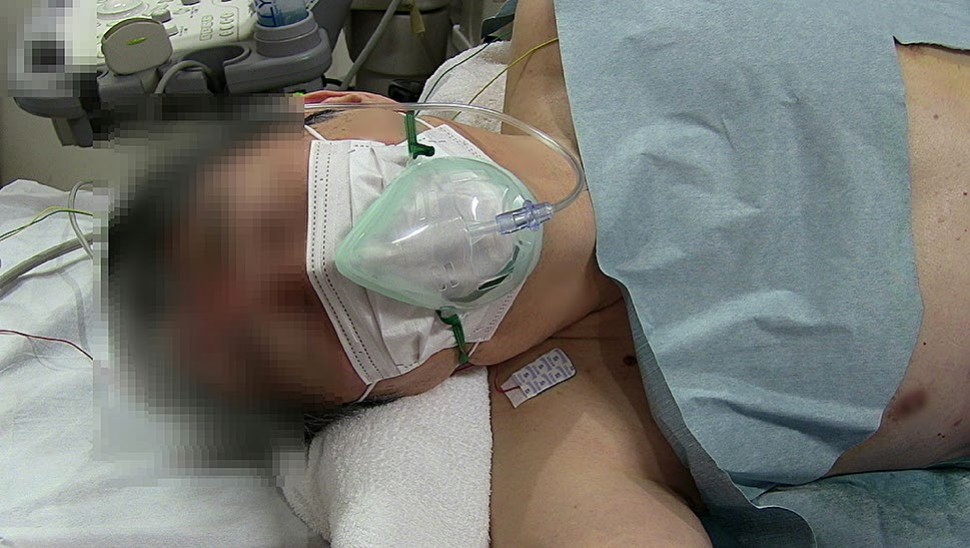
Patients also should wear facemasks, and oxygen masks should be on top

Ablation itself is not an aerosol generating procedure (Figs. 1, 2, 3) [39]. Ablation is generally performed with local anesthesia. In some institutions, ablation is performed with general anesthesia, and general anesthesia is an aerosol generating procedure. When general anesthesia is performed, ablation rooms are considered as high-risk areas of infection.

### Vascular interventions

Vascular interventions, such as transarterial chemoembolization and hepatic arterial infusion chemotherapy, are recommended for cases of unresectable, large/multifocal HCCs without vascular invasion nor extrahepatic spread. Vascular interventions are cytoreductive treatments in most cases. Therefore, they may be suspended until they can be performed safely with sufficient resources.

Vascular interventions should be suspended in cases of risk of decompensation or comorbidities that increase the risk of severe COVID-19 because of reduced inpatient beds for post-procedure care. If vascular intervensions are performed, selective or super selective chemoembolization should be attempted to prevent severe postembolization symptoms.

Vascular interventions, themselves are not aerosol generating procedures [39].

### Radiation therapy

Radiation therapy, including proton beam and carbon ion beam therapies, can be used as an option for local control of HCC. Palliative care for cases receiving the therapy to control symptoms or at low risk of progression is better to delay the schedule of radiation [40]. However, radiation therapy for patients with rapidly progressing HCC may outweigh the risks of the novel coronavirus infection. Radiation therapy for function- or life-threatening situation such as spinal cord compression and inferior vena cava syndrome have to be treated without delay. The course of radiation should be shortened, for example, single-fraction treatment for bone pain, and less fractionation when appropriate.

### Systemic therapy

Systemic therapy is for advanced-stage HCC such as with macrovascular invasion or extrahepatic metastasis and with Child–Pugh class A liver function. For cases of advanced HCC treated with systemic therapy, oral tyrosine kinase inhibitors would be better than infusional regimens during the pandemic to protect both patients and medical staff. If available, video call should be used to manage common adverse events. Intravenous chemotherapy should be administered in a dedicated section of outpatient service. The impact of immunotherapy on the course of COVID-19 is not known since there is no sufficient data. Immunotherapy should be considered on a case-by-case basis. Clinical trial recruitment should be suspended until the COVID-19 pandemic settles down. During the COVID-19 pandemic, postponing of locoregional therapies may be necessary. Oral targeted drugs might be a substitute for some patients.

## Follow-up for HCC

It is recommended to follow up the patients by telemedicine or online consultation to minimize hospital visits in the circumstances of COVID-19 pandemic. However, due to its high frequency of the recurrence in HCC, imaging examination to detect recurrence at the early stage should not be postponed.

## Conclusions

In these difficult times, it is our obligation to preserve high management standards of other diseases as well as to effectively handle the COVID-19 pandemic in spite of the shortage of medical resources, such as personnel, beds, PPE and others. To keep our patients and ourselves safe, we should share our experiences and strategies to manage our patients.

The novel coronavirus infection will not settle down in the near future. We need to collect real-world data to mitigate the impact of COVID-19. It is mandatory to collect follow-up data of patients who recovered from COVID-19. There have not been any data available on COVID-19 from long-term viewpoint, since COVID-19 was first reported at the end of 2019. It is important to determine the symptomatic and asymptomatic incidence of the novel coronavirus infection by large-scale serological testing not only in the general population but also in patients with HCC and to survey their COVID-19-related morbidity and mortality to adjust management strategies of HCC.

## Abbreviations

COVID-19: Coronavirus disease 2019
PPE: Personal protective equipment
HCC: Hepatocellular carcinoma
HBV: Hepatitis B virus
HCV: Hepatitis C virus
CDC: Centers for Disease Control and Prevention
US: Ultrasound
AFP: Alpha-fetoprotein
